# Exploring Women’s Knowledge of Nutrition and Bone Health: A Preventive Focus on Osteoporosis

**DOI:** 10.7759/cureus.83722

**Published:** 2025-05-08

**Authors:** Silpa Chintham, Malathi S, Panneerselvam Periasamy, Sivakumar Gopalakrishnan

**Affiliations:** 1 Department of Community Health Nursing, Vinayaka Missions Annapoorana College of Nursing, Vinayaka Mission’s Research Foundation (Deemed to be University), Chittoor, IND; 2 Community Health Sciences, Vinayaka Missions Annapoorana College of Nursing, Salem, IND; 3 Department of Physiology, Government Erode Medical College, Perundurai, IND; 4 Department of Pathophysiology, American university of Antigua, Antigua, ATG

**Keywords:** awareness, dietary factors, osteoporosis, south india, women

## Abstract

Background

Osteoporosis is a chronic skeletal disorder characterized by decreased bone mineral density and deterioration of bone microarchitecture, resulting in increased bone fragility and fracture risk. Among the modifiable risk factors, diet and lifestyle play a critical role in both the prevention and progression of osteoporosis. Perimenopausal women are particularly susceptible to bone loss due to hormonal changes, making it imperative to evaluate their awareness of dietary influences on bone health. This study aimed to assess the level of awareness regarding osteoporosis-related dietary factors among perimenopausal women and to identify key knowledge gaps that warrant targeted educational interventions.

Methods

A cross-sectional study was conducted among 200 perimenopausal women using a convenience sampling technique. A structured, validated 25-item questionnaire was administered to evaluate awareness of dietary factors associated with osteoporosis. Each correct response scored one point. Based on the total score, awareness was categorized as inadequate (0-12), moderately adequate (13-19), or adequate (20-25). Data were analyzed using descriptive statistics, including means, standard deviations, frequencies, and percentages. Item-wise analysis was carried out to evaluate specific areas of knowledge. The Chi-square (χ²) test was used to assess associations between awareness levels and selected demographic variables, including age, education, occupation, socioeconomic status, diet type, and family history of osteoporosis. A p-value of <0.05 was considered statistically significant.

Results

The mean age of participants was 51.26 ± 7.12 years. Of the 200 women surveyed, 133 (66.5%) exhibited inadequate awareness, 67 (33.5%) showed moderately adequate awareness, and none (0.0%) achieved the adequate awareness level. The overall mean awareness score was 10.35 ± 3.31. Item-wise analysis revealed relatively better awareness in certain areas; 176 (88.0%) participants correctly identified the critical age group for bone health, and 166 (83.0%) recognized sunlight as a major source of vitamin D. However, significant knowledge gaps were observed in essential domains: only 74 (37.0%) knew calcium-rich food sources, 80 (40.0%) were aware of the negative impact of soft drinks on bone health, and just 62 (31.0%) understood the importance of osteoporosis-related health education. A statistically significant association (p = 0.001) was found between awareness levels and several demographic factors, including age, educational status, occupation, socioeconomic background, diet type, and family history of osteoporosis.

Conclusion

The study highlights a generally low level of awareness regarding dietary factors related to osteoporosis among perimenopausal women, with critical deficiencies in knowledge of essential preventive strategies. These findings call for the implementation of targeted, community-based educational programs to enhance dietary literacy and bone health awareness. Furthermore, the strong association between awareness and demographic characteristics underscores the need for tailored interventions that address the specific needs of vulnerable subgroups. Incorporating osteoporosis education into routine women’s health services may significantly contribute to the early prevention and management of this silent disease.

## Introduction

Osteoporosis is a skeletal disorder marked by reduced bone density and deterioration of bone tissue structure, resulting in weakened bone integrity and an elevated susceptibility to fractures due to fragility [1]. Osteoporosis may manifest as primary or secondary, with primary osteoporosis being the most common. It is frequently observed in postmenopausal women, known as postmenopausal osteoporosis. Secondary osteoporosis is a complication of an underlying condition. Various factors have been associated with an elevated risk of osteoporosis. Conversely, there are several strategies known to enhance bone mineral density and decrease the likelihood of fractures. Osteoporosis is often undiagnosed, yet prevention is preferable to treatment, and osteoporosis is a preventable ailment; the initial step in prevention is raising awareness of the risk factors [2].

Osteoporosis impacts people of all ages, but it is most prevalent among postmenopausal women and the elderly [3]. Females face a 30-50% chance of experiencing fractures related to osteoporosis throughout their lifetime [4]. Osteoporosis affects 21.7% of the elderly worldwide, with Asia having the highest rate at 24.3%, followed by Europe at 16.7%, and America at 11.5%. Additionally, osteoporosis is prevalent in 35.3% of older women and 12.5% of older men globally [5]. The estimated number of individuals suffering from osteoporosis in Indian communities is expected to reach 26 million, with a forecasted rise to 36 million by 2013. The likelihood of experiencing osteoporotic fractures during one's lifetime is 30-50% for women and 15-30% for men [6]. Lifestyle choices and dietary habits play a crucial role in maintaining optimal bone health. Inadequate intake of calcium and vitamin D can result in alterations in bone structure and strength. Consuming sufficient dietary calcium is closely associated with higher bone mineral density throughout the body [7].

Osteoporosis can be influenced by lifestyle choices such as lack of physical activity and poor diet, as well as factors beyond our control, such as gender, getting older, and genetic predisposition [8]. Lifestyle modifications and, in some instances, medication can help reduce the risks associated with osteoporosis; individuals with osteoporosis may receive a combination of treatments. Dietary adjustments, physical activity, and measures to prevent falls are all forms of lifestyle modifications [9]. Additionally, prior to being diagnosed, 80% of women with osteoporosis were not aware of their risk.

In recent years, more attention has been given to previously underappreciated risk factors and preventive strategies in osteoporosis management. Genetic predisposition continues to be a significant risk factor, with recent studies identifying various genetic markers that may increase susceptibility to osteoporosis [10-13]. Additionally, the role of chronic inflammation and certain autoimmune diseases has been linked to increased bone resorption, accelerating osteoporosis progression [11]. Preventive measures have also expanded beyond traditional calcium and vitamin D supplementation, with emerging evidence highlighting the role of magnesium and vitamin K2 in improving bone health [12]. Resistance training and weight-bearing exercises have also been shown to improve bone density, particularly in postmenopausal women. Moreover, newer pharmacological treatments, such as the introduction of sclerostin inhibitors, have provided promising results in reducing fracture risk among high-risk populations [13]. Therefore, it is essential to increase awareness of osteoporosis risk factors and methods to enhance bone health [6]. The objectives of the study were to assess the awareness of dietary factors related to osteoporosis among women and to examine the association between the level of awareness and selected demographic variables.

## Materials and methods

Study design and setting

The present study utilized a cross-sectional research design to evaluate the awareness of dietary factors associated with osteoporosis among women aged 31 to 60 years. The study was conducted over a period from October 1, 2020, to March 1, 2022, at Annapoorna College of Nursing, Chennai, Tamil Nadu, following approval from the Institutional Ethics Committee (Ethical Clearance No VMACON/IEC/01/2020 dated 29/09/2020). A cross-sectional approach was chosen to capture data at a single point in time, providing a snapshot of the participants' awareness levels concerning osteoporosis-related dietary factors. The selected areas represented a diverse socioeconomic background to ensure variability in educational attainment and access to healthcare services among the participants.

Selection criteria

**Figure 1:**
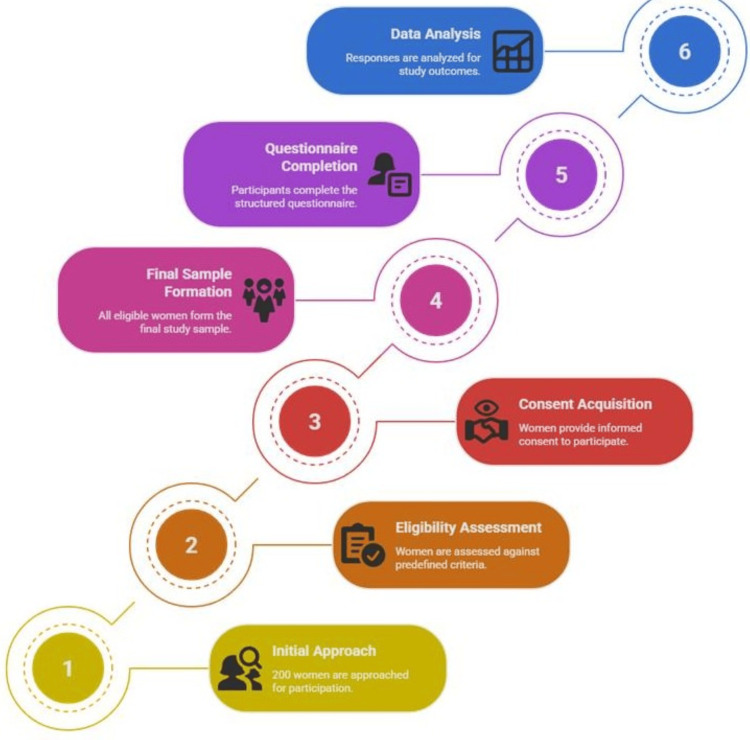
Participant Selection Process

The inclusion criteria for this study were women aged 31 to 60 years who were available and willing to participate during the data collection period. Participants were required to provide informed consent prior to participation. Women with a prior diagnosis of osteoporosis or with coexisting chronic conditions that might influence dietary intake or awareness were excluded to avoid confounding results. Additionally, individuals who declined participation were excluded from the sample. The final sample comprised 200 women, selected using a non-probability convenient sampling technique, which allowed for the inclusion of participants based on their availability and willingness to participate (Figure 1).

Sample size calculation

The sample size for the present study was calculated based on the assumption of a 50% expected prevalence of awareness regarding dietary factors related to osteoporosis, considering the lack of prior regional data. This conservative estimate was chosen to yield the maximum sample size. Using a 95% confidence level and a 7% margin of error, the minimum required sample size was determined to be approximately 196 participants. To account for possible non-responses or incomplete data, the final sample was rounded up to 200 women. Furthermore, a post hoc power analysis conducted using G*Power software confirmed that with a sample size of 200 and a moderate effect size (w = 0.3), the statistical power exceeded 90% for Chi-square tests of independence. This indicates a high probability of detecting significant associations between awareness levels and demographic variables, thereby supporting the reliability and validity of the study findings.

Dietary factors assessed

The study specifically explored dietary factors known to influence bone health and osteoporosis risk. The structured questionnaire assessed participants’ awareness of key nutrients such as calcium, vitamin D, and protein, along with their knowledge of dietary sources rich in these nutrients (e.g., milk, cheese, yogurt, green leafy vegetables, nuts, and fish). In addition, awareness related to the role of sunlight in vitamin D synthesis, the effect of excessive caffeine and carbonated soft drink consumption, and the importance of dietary diversity in maintaining bone health were also evaluated. These factors were selected based on established clinical and public health recommendations regarding osteoporosis prevention and nutritional adequacy.

Data sources and variables

Data collection involved face-to-face interviews using a structured questionnaire tailored for this study, comprising 25 multiple-choice questions focused on dietary factors affecting osteoporosis. Key topics included calcium and vitamin D intake, dairy consumption, and other relevant dietary practices. Alongside knowledge-based questions, demographic data such as age, education level, occupation, and dietary restrictions were gathered.

Questionnaire development and validation

The questionnaire’s development involved a thorough validation process. Initially, a pilot test was conducted with 30 participants not included in the final study. Reliability was assessed using SPSS version 25, yielding a Cronbach’s alpha of 0.89, which indicates high internal consistency. Content validity was established through consultations with experts in nutrition and osteoporosis management, ensuring the questionnaire’s relevance and appropriateness for the cultural context of the population studied. The primary variables examined were participants’ awareness of dietary factors related to osteoporosis and their demographic characteristics.

Scoring methodology and categorization of awareness levels

In this study, the participants’ awareness levels regarding dietary factors associated with osteoporosis were assessed using a structured questionnaire comprising 25 items (Appendices), with each correct response assigned one point, resulting in a maximum possible score of 25. The scoring thresholds for categorizing overall awareness levels were initially determined through expert consultation and aligned with methodologies reported in similar awareness-based studies. Awareness scores were classified as follows: inadequate (0-12), moderately adequate (13-19), and adequate (20-25). However, within the present study cohort, no participant achieved a score falling within the “adequate” category, with all responses distributed between the “inadequate” and “moderately adequate” levels. This outcome suggests that the expert-defined scoring criteria may not fully reflect the actual awareness distribution in the population. This structured scoring approach nonetheless enabled a systematic assessment of the participants’ knowledge and helped identify key gaps requiring targeted educational interventions.

Statistical analysis

Once collected, the data were analyzed using both descriptive and inferential statistical methods. Descriptive statistics, including frequency, percentage distribution, mean, and standard deviation, were used to summarize the participants' levels of awareness regarding dietary factors of osteoporosis. The demographic variables were also summarized using descriptive measures. To examine potential associations between the level of awareness and demographic characteristics (such as age, education level, and occupation), inferential statistics were applied. Chi-square tests were employed to assess these relationships, with a significance level set at P < 0.05. All analyses were performed using SPSS software version 25.0.

Ethical considerations

Ethical approval for the study was obtained from the Institutional Ethics Committee before the commencement of the study. Written informed consent was obtained from all participants after explaining the purpose of the study, ensuring that participation was voluntary, and that the data would be handled confidentially. Participants were given the right to withdraw from the study at any stage without any consequences.

## Results

Table 1 describes the frequency and percentage distribution of demographic variables among women included in the study. The data show that the majority of participants, 114 (57%), were in the age group of 41-50 years, followed by 49 (24.5%) in the age group of 31-40 years, and 37 (18.5%) in the 51-55 years and above category. Regarding educational status, 76 (38%) of the women had a high school education, 67 (33.5%) had higher secondary education, 42 (21%) had no formal education, and only 15 (7.5%) were graduates.

**Table 1:** Frequency- and percentage-wise distribution of demographic variables among women

Demographic Variable	Frequency N (%)
Age in years	
31-40 years	49 (24.5%)
41-50 years	114 (57%)
51-55 years and above	37 (18.5%)
Education	
No formal education	42 (21%)
High school	76 (38%)
Higher secondary	67 (33.5%)
Graduate	15 (7.5%)
Occupation	
Working women	89 (44.5%)
Homemaker	111 (55.5%)
Socioeconomic status	
Lower class	97 (48.5%)
Middle class	81 (40.5%)
Upper class	22 (11%)
Type of diet	
Vegetarian	62 (31%)
Mixed	138 (69%)
Previous history of osteoporosis in family	
Present	43 (21.5%)
Absent	157 (78.5%)

The occupation status of the participants revealed that 111 (55.5%) were homemakers, while 89 (44.5%) were working women. In terms of socioeconomic status, nearly half of the participants, 97 (48.5%), belonged to the lower class, 81 (40.5%) were from the middle class, and 22 (11%) were from the upper class. The type of diet consumed by the women showed that 138 (69%) had a mixed diet, while 62 (31%) were vegetarians. Additionally, 43 (21.5%) of the women reported a previous history of osteoporosis in their family, while 157 (78.5%) did not have any family history of the condition (Table 1).

The descriptive statistics of the item-wise correct response rates are summarized in Table 2. The average correct response rate for all 19 awareness items was 65.89% (SD = 17.27%), reflecting moderate awareness with notable variability among participants. The correct response rates ranged from a minimum of 31.0% to a maximum of 88.0%. The 25th percentile (Q1) was 57.75%, the median (50th percentile) was 67.0%, and the 75th percentile (Q3) reached 78.25%. These statistics highlight the uneven distribution of knowledge across different dietary factors, emphasizing the presence of significant knowledge gaps in certain domains despite relatively higher awareness in others.

**Table 2:** Descriptive Statistics of Awareness Scores

Statistic	Value (%)
Count	19
Mean ± SD	65.89 ±17.27
Minimum	31.00
25th percentile (Q1)	57.75
50th percentile (Median)	67.00
75th percentile (Q3)	78.25
Maximum	88.00

Table 3 presents the association between the level of awareness of dietary factors related to osteoporosis and selected demographic variables. A statistically significant association was observed between age and awareness level (p = 0.001). Women aged 41-50 years demonstrated better awareness, with 72 (54.1%) showing inadequate awareness and 42 (62.7%) showing moderately adequate awareness. Educational status was also significantly associated with awareness levels (p = 0.001); 52 (77.6%) of women with higher secondary education and 15 (22.4%) graduates had a higher level of awareness compared to those with no formal or only high school education. Occupational status showed a significant association as well (p = 0.001), with all 67 (100%) homemakers demonstrating moderately adequate awareness, while 89 (66.9%) of working women had inadequate awareness.

**Table 3:** Association between the level of awareness of dietary factors of osteoporosis among women with their selected demographic variables Note: N = 200 (Used for percentage calculations). *p < 0.05 Significant **p < 0.001 Highly significant

Demographic Variables	Level of Awareness		Chi-square (X^2^)	p-value
	Inadequate level of awareness	Moderately adequate level of awareness		
	N (%)	N (%)		
Age in years				
31-40 years	49 (36.8%)	0 (0%)	44.5	0.001**
41-50 years	72 (54.1%)	42 (62.7%)		
51-55 years and above	12 (9.1%)	25 (37.3%)		
Educational Status				
No formal education	42 (31.6%)	0 (0)	147.7	0.001**
High school education	76 (57.1%)	0 (0)		
Higher secondary school education	15 (11.3%)	52 (77.6%)		
Graduates	0 (0)	15 (22.4%)		
Occupation Status				
Employed	89 (66.9%)	0 (0)	80.7	0.001**
Housemaker	44 (33.1%)	67 (100%)		
Socioeconomic Status				
Lower class	97 (72.9%)	0 (0)	110.2	0.001**
Middle class	36 (27.1%)	45 (67.2%)		
Upper class	0 (0)	22 (32.8%)		
Type of diet				
Vegetarian	62 (46.6%)	0 (0)	45.26	0.001**
Mixed diet	71 (53.4%)	67 (100%)		
Previous History of Osteoporosis in Family				
Present	43 (32.3%)	0 (0)	25.5	0.005*
Absent	90 (67.7%)	67 (100%)		

Socioeconomic status also exhibited a significant correlation with awareness levels (p = 0.001). Among women from the lower socioeconomic group, 97 (72.9%) had inadequate awareness, whereas 45 (67.2%) from the middle class and 22 (32.8%) from the upper class showed moderately adequate awareness. A similar pattern was seen in dietary habits, where women with a mixed diet (71; 53.4%) had better awareness compared to vegetarians (62; 46.6%) (p = 0.001). Lastly, a significant association was found between family history of osteoporosis and awareness (p = 0.005), with 90 (67.7%) of women without a family history showing moderately adequate awareness, compared to 43 (32.3%) with a family history (Table 2).

Item-wise awareness analysis of dietary factors related to osteoporosis

In addition to evaluating the overall awareness levels, an item-wise analysis of the responses to individual questionnaire items was performed to assess the specific knowledge gaps among the study participants. Table 4 presents the frequency distribution of correct and incorrect responses for each of the 19 key awareness questions included in the structured questionnaire, along with the corresponding Chi-square (X^²^) values and p-values.

**Table 4:** Analysis of Individual Questionnaire Items on Awareness of Dietary Factors for Osteoporosis

Q. No.	Knowledge Area	Correct Responses (n=200)	Incorrect Responses (n=200)	Correct Responses (%)	Chi-square (X²)	p-value
7	Relation of osteoporosis	162	38	81	5.41	0.003
8	Essential mineral for bone	111	89	55.5	24.4	0.031
9	Role of Vitamin D	152	48	76	21.65	0.009
10	Calcium-rich food	74	126	37	9.25	0.004
11	Sunlight as Vitamin D source	166	34	83	8.64	0.047
12	High-risk group for osteoporosis	131	69	65.5	8.67	0.048
13	Recommended calcium intake	120	80	60	11.08	0.041
14	Effect of soft drinks	80	120	40	15.5	0.016
15	Most beneficial dairy product	162	38	81	13.64	0.006
16	Consequences of calcium deficiency	142	58	71	10.82	0.035
17	Calcium-rich leafy greens	146	54	73	17.24	0.023
18	Source of Vitamin K2	134	66	67	7.79	0.007
19	Effect of high caffeine intake	134	66	67	10.84	0.025
20	Calcium supplements with Vitamin D	147	53	73.5	12.33	0.003
21	Critical age group for bone health	176	24	88	14.12	0.046
22	Calcium-rich nuts	159	41	79.5	20.7	0.014
23	Importance of calcium in postmenopausal women	163	37	81.5	8.99	0.033
24	Awareness of foods preventing osteoporosis	83	117	41.5	15.28	0.016
25	Received health education on bone health	62	138	31	16.85	0.026

An item-wise analysis of the questionnaire responses was performed to identify specific knowledge gaps related to dietary factors associated with osteoporosis. The percentage of correct responses for each item is presented in Table 4, along with corresponding Chi-square values and p-values. The analysis revealed considerable variation in awareness across different knowledge areas. The highest correct response rates were recorded for items related to the critical age group for bone health (88%), sunlight as a natural source of vitamin D (83%), and the association between osteoporosis and bone health (81%). Conversely, the lowest awareness was observed for questions concerning formal health education on bone health (31%), identification of foods that help prevent osteoporosis (41.5%), knowledge of calcium-rich foods (37%), and understanding the negative impact of soft drink consumption on calcium absorption (40%). These findings highlight specific areas where participants demonstrated considerable knowledge gaps (Table 5).

**Table 5:** Item-wise Awareness Analysis of Dietary Factors

Question No.	Knowledge Area	Correct Responses (%)	Brief Description
Item-wise Awareness Analysis of Dietary Factors 7	Relation of osteoporosis	81	High awareness of osteoporosis as a bone-related condition.
8	Essential mineral for bone	55.5	Moderate awareness regarding calcium as an essential mineral for bones.
9	Role of Vitamin D	76	Good understanding of the role of Vitamin D in calcium absorption.
10	Calcium-rich food	37	Low identification of calcium-rich foods like dairy and green vegetables.
11	Sunlight as Vitamin D source	83	High awareness of sunlight as a key Vitamin D source.
12	High-risk group for osteoporosis	65.5	Moderate understanding of the high-risk group being postmenopausal women.
13	Recommended calcium intake	60	Moderate knowledge about recommended calcium intake levels.
14	Effect of soft drinks	40	Poor awareness of the negative impact of soft drinks on calcium absorption.
15	Most beneficial dairy product	81	Good awareness of dairy products beneficial for bone health.
16	Consequences of calcium deficiency	71	Moderate understanding of the effects of calcium deficiency on bone weakening.
17	Calcium-rich leafy greens	73	Moderate awareness about leafy greens high in calcium.
18	Source of Vitamin K2	67	Limited knowledge about Vitamin K2 sources.
19	Effect of high caffeine intake	67	Limited understanding of the harmful effects of high caffeine intake on calcium absorption.
20	Calcium supplements with Vitamin D	73.5	Good awareness about the need for Vitamin D with calcium supplements.
21	Critical age group for bone health	88	Highest awareness regarding age groups needing bone health attention.
22	Calcium-rich nuts	79.5	Good knowledge of nuts as a source of calcium.
23	Importance of calcium in postmenopausal women	81.5	Good awareness of the importance of calcium in postmenopausal women.
24	Awareness of foods preventing osteoporosis	41.5	Poor knowledge regarding osteoporosis-preventive foods.
25	Received health education on bone health	31	Lowest awareness regarding receiving formal health education on bone health.

The application of the Chi-square test further assessed the association between awareness of individual dietary factors and demographic characteristics. Several items demonstrated statistically significant associations (p < 0.05) with demographic variables. Notably, significant associations were found for awareness regarding calcium-rich foods (p = 0.004), the role of vitamin D (p = 0.009), recommended calcium intake (p = 0.041), and the effect of soft drink consumption on calcium absorption (p = 0.016).

Overall, these findings emphasize that while participants demonstrated reasonably good awareness of certain osteoporosis-related dietary factors, substantial gaps persist, particularly concerning preventive strategies and health education. This analysis highlights priority areas for targeted educational interventions aimed at enhancing osteoporosis prevention awareness among women.

## Discussion

This study employed a cross-sectional research design to evaluate the awareness of dietary factors associated with osteoporosis among perimenopausal women in selected areas of Chittoor, South India. The structured questionnaire, consisting of 25 validated items, was designed to assess participants’ knowledge on various aspects of osteoporosis prevention, particularly focusing on dietary sources of calcium, the role of vitamin D, and the impact of lifestyle factors such as soft drink and caffeine consumption. To ensure clarity and reproducibility, a defined scoring system was implemented wherein each correct response was awarded one point, and awareness levels were categorized as inadequate (0-12 points), moderately adequate (13-19 points), and adequate (20-25 points). This scoring threshold was determined through expert consultation and is consistent with methodologies used in similar awareness studies [14-19].

The findings revealed that the mean awareness score was 10.35 ± 3.31, indicating limited overall knowledge about osteoporosis-related dietary factors among the study participants. Notably, none of the women achieved the “adequate” awareness category, with the distribution confined to 66.5% demonstrating inadequate awareness and 33.5% exhibiting moderately adequate awareness. These results are concerning, as inadequate awareness may contribute to the continued high prevalence of osteoporosis-related complications in this demographic group. Comparable observations were reported by Chan et al. (2019), who found that while general knowledge about bone health was relatively high, specific awareness related to osteoporosis prevention remained suboptimal [14]. Similarly, Pouresmaeili et al. (2018) [1] and Hannan et al. (2000) [15] emphasized the pivotal role of calcium and vitamin D intake in maintaining bone mineral density and reducing fracture risk, yet highlighted the persistence of low awareness levels in many populations [1,15].

The item-wise analysis of the questionnaire responses provided valuable insights into the specific knowledge domains where awareness was either adequate or lacking. Higher correct response rates were observed for questions related to the critical age group for bone health (88%), the role of sunlight as a source of vitamin D (83%), and the relation of osteoporosis to bone health (81%). These findings suggest that while general conceptual awareness of osteoporosis exists, detailed knowledge about preventive dietary strategies remains insufficient. In particular, questions regarding receiving formal health education on bone health (31%), awareness of calcium-rich foods (37%), and the effects of soft drink consumption on calcium absorption (40%) had the lowest correct response rates, underscoring significant gaps in understanding. Such gaps are particularly concerning because these dietary and lifestyle factors are modifiable and play a crucial role in osteoporosis prevention.

The descriptive statistics of the item-wise analysis further reinforced these findings, with a mean correct response rate of 65.89% and a standard deviation of 17.27%, reflecting considerable variability across the knowledge items. The awareness scores ranged from 31% to 88%, highlighting the uneven distribution of knowledge among the respondents. The median (50th percentile) score of 67% and the 75th percentile of 78.25% suggest that while some participants possess moderate awareness, a substantial proportion remain poorly informed.

The Chi-square test of independence was employed to examine the relationship between awareness levels and selected demographic variables, including age, educational status, occupational status, socioeconomic status, type of diet, and family history of osteoporosis. The analysis revealed statistically significant associations (p = 0.001), indicating that demographic factors strongly influenced awareness levels. These findings are in agreement with previous studies, such as that of Föger-Samwald et al. (2020), who demonstrated that both age and education are key determinants of osteoporosis-related knowledge [2]. Agrawal et al. (2023) also reported that women from lower socioeconomic backgrounds tend to have lower awareness levels, emphasizing the role of educational and financial disparities in health literacy [6]. Additionally, similar associations between higher educational attainment and improved osteoporosis awareness have been consistently reported in the literature [18, 19].

The item-wise Chi-square analysis further highlighted specific areas where demographic variables significantly influenced awareness. For example, significant associations were noted between demographic factors and awareness regarding calcium-rich foods (p = 0.004), the role of vitamin D (p = 0.009), recommended calcium intake (p = 0.041), and the impact of soft drink consumption on calcium absorption (p = 0.016). These findings suggest that improving awareness in these domains could be effectively achieved through targeted educational interventions, particularly for women belonging to lower educational and socioeconomic strata.

The overall findings of this study align with earlier research that underscores the importance of health education in enhancing osteoporosis-related knowledge. Ward and Klesges (2001) highlighted the role of lifestyle modifications, including weight-bearing exercises and calcium-rich diets, in osteoporosis prevention [16]. Furthermore, Al-Muraikhi et al. (2017) demonstrated that well-structured educational programs significantly improved women's knowledge and attitudes toward osteoporosis prevention [17]. These findings support the need for community-based awareness campaigns, interactive health education sessions, and culturally sensitive materials tailored to the needs of the target population.

The lack of formal health education, as indicated by the lowest correct response rate for that item (31%), further emphasizes the need for organized awareness initiatives. These programs should not only focus on providing knowledge but also address socio-cultural barriers, such as misconceptions about dietary sources of calcium and the role of vitamin D, which may hinder the adoption of healthy behaviors.

In light of these results, the study recommends the implementation of structured educational interventions focusing on critical knowledge domains, including calcium and vitamin D intake, harmful dietary practices (such as excessive soft drink and caffeine consumption), and the significance of early preventive strategies. Involving local healthcare providers, women's self-help groups, and community health workers could enhance the effectiveness of these interventions, ensuring better reach and impact.

Overall, this study contributes to the existing body of literature by offering a detailed item-wise analysis of osteoporosis-related dietary awareness and by identifying demographic predictors of knowledge levels. The findings underscore the urgent need for targeted health promotion activities aimed at bridging the knowledge gaps among women, particularly in low-resource settings where osteoporosis awareness remains critically low.

Strengths

One of the key strengths of this study is its structured approach to assessing awareness using a validated questionnaire, which allowed for both overall scoring and detailed item-wise analysis of osteoporosis-related dietary knowledge. The use of a clearly defined scoring system with categorized thresholds (inadequate, moderately adequate, and adequate awareness levels) enhanced the objectivity, transparency, and reproducibility of the findings. Additionally, the inclusion of descriptive statistics and Chi-square analysis provided robust insights into the relationship between awareness levels and various demographic factors, such as age, educational status, occupation, socioeconomic status, dietary habits, and family history of osteoporosis. The item-wise breakdown of correct response rates offered a more granular understanding of specific knowledge gaps, identifying critical areas where targeted educational interventions are needed. This comprehensive analytical approach contributes valuable data for developing public health strategies aimed at improving osteoporosis awareness among women in similar demographic settings.

Limitations

Despite these strengths, the study has certain limitations that should be acknowledged. First, the cross-sectional design limits the ability to establish causal relationships between awareness levels and osteoporosis-related behaviors or outcomes. Longitudinal studies would be required to assess how awareness translates into preventive practices over time. Second, the use of convenience sampling may introduce selection bias and affect the generalizability of the findings, as the sample may not fully represent the broader population of perimenopausal women in different geographic or socioeconomic contexts. Third, the study primarily focused on awareness regarding dietary factors, excluding other important contributors to bone health such as physical activity, hormonal factors, and genetic predisposition. Additionally, the assessment relied on self-reported responses, which could be influenced by recall bias or social desirability bias, potentially leading to over- or underestimation of actual knowledge levels.

Furthermore, while the questionnaire was validated and pilot-tested, there remains a possibility that certain questions may not fully capture the participants' depth of understanding, especially in populations with limited health literacy. Future studies should consider including qualitative assessments, such as focus group discussions or in-depth interviews, to complement quantitative findings and provide deeper insights into the reasons behind knowledge gaps.

Overall, these limitations suggest the need for further research using larger, randomly selected samples and mixed-method approaches to enhance the reliability and applicability of the results across diverse populations.

## Conclusions

The study reveals significant gaps in women's awareness of dietary factors related to osteoporosis, particularly regarding calcium and vitamin D intake and the adverse effects of unhealthy dietary habits. These findings highlight the need for targeted public health initiatives to improve osteoporosis prevention.

Community-based educational programs focused on osteoporosis risk factors and prevention, especially for women from lower educational and socioeconomic backgrounds, are essential. Collaboration with healthcare providers and local health agencies to conduct culturally appropriate workshops and awareness campaigns can enhance outreach and effectiveness.

Empowering healthcare professionals through training in nutritional education can support the wider dissemination of osteoporosis-related knowledge. These professionals can play a vital role in patient education and the promotion of healthy practices.

Future research should evaluate the long-term impact of such interventions on improving awareness and preventive behaviors, supporting the development of effective public health strategies to reduce osteoporosis risk and improve outcomes among vulnerable populations.

## Appendices

Questionnaire on awareness of dietary factors related to osteoporosis

Part I: Demographic Information

**Table 6:** Part I: Demographic Information Credits: Silpa Chintham, Malathi S, Panneerselvam Periasamy, Sivakumar Gopalakrishnan

Question	Options
1. Age Group	☐ 31–40 years ☐ 41–50 years ☐ 51–55 years and above
2. Educational Status	☐ No formal education ☐ High school ☐ Higher secondary ☐ Graduate
3. Occupation	☐ Homemaker ☐ Working
4. Socio-economic Status	☐ Lower class ☐ Middle class ☐ Upper class
5. Type of Diet	☐ Vegetarian ☐ Mixed
6. Family History of Osteoporosis	☐ Present ☐ Absent

Part II: Awareness of Dietary Factors Related to Osteoporosis

(Choose the correct option. Only one answer is correct for each.)

**Table 7:** Awareness of Dietary Factors Related to Osteoporosis Credits: Silpa Chintham, Malathi S, Panneerselvam Periasamy, Sivakumar Gopalakrishnan

Question	Options
7. Osteoporosis is related to:	☐ Heart disease ☐ Bone health ☐ Skin condition ☐ Lung infection
8. Which mineral is most essential for bone strength?	☐ Iron ☐ Calcium ☐ Potassium ☐ Zinc
9. Vitamin D helps in the absorption of:	☐ Iron ☐ Glucose ☐ Calcium ☐ Sodium
10. Which of the following is a good source of calcium?	☐ Bananas ☐ Milk ☐ Rice ☐ Tomatoes
11. Sunlight is a natural source of:	☐ Vitamin A ☐ Vitamin D ☐ Vitamin K ☐ Vitamin C
12. Which group is at higher risk of osteoporosis?	☐ Children ☐ Young men ☐ Postmenopausal women ☐ Teenagers
13. What is the recommended dietary calcium intake for adults (approx.)?	☐ 200 mg/day ☐ 500 mg/day ☐ 1000 mg/day ☐ 3000 mg/day
14. Regular intake of carbonated soft drinks may:	☐ Improve bone strength ☐ Have no effect ☐ Decrease calcium absorption ☐ Increase calcium levels
15. Which dairy product is most beneficial for bone health?	☐ Ice cream ☐ Butter ☐ Cheese ☐ Milk
16. A calcium-deficient diet can lead to:	☐ Hair loss ☐ Bone weakening ☐ Tooth decay ☐ None of the above
17. Which leafy green is high in calcium?	☐ Spinach ☐ Cabbage ☐ Mint ☐ Lettuce
18. Vitamin K2, helpful for bones, is found in:	☐ Butter ☐ Natto (fermented soy) ☐ Apples ☐ Coffee
19. High caffeine intake may:	☐ Increase bone density ☐ Reduce bone loss ☐ Enhance calcium absorption ☐ Reduce calcium absorption
20. Calcium supplements should ideally be taken with:	☐ Water only ☐ A high-fat meal ☐ Vitamin D ☐ On an empty stomach
21. Which age group should pay special attention to bone health?	☐ 0–10 years ☐ 20–30 years ☐ 40 years and above ☐ All of the above
22. Which of the following nuts is rich in calcium?	☐ Cashews ☐ Almonds ☐ Walnuts ☐ Peanuts
23. Do you think calcium intake is important in postmenopausal women?	☐ Yes ☐ No
24. Are you aware of foods that can prevent osteoporosis?	☐ Yes ☐ No
25. Have you received any health education on diet and bone health?	☐ Yes ☐ No
